# Corticosterone in feathers of laying hens: an assay validation for evidence-based assessment of animal welfare

**DOI:** 10.1016/j.psj.2020.06.065

**Published:** 2020-07-24

**Authors:** K.E. Häffelin, R. Lindenwald, F. Kaufmann, S. Döhring, B. Spindler, R. Preisinger, S. Rautenschlein, N. Kemper, R. Andersson

**Affiliations:** ∗ Faculty of Agriculture Sciences and Landscape Architecture, Osnabrück University of Applied Sciences, 49090 Osnabrück, Germany; † Clinic for Poultry, University of Veterinary Medicine Hannover (Foundation), 30559 Hannover, Germany; ‡ Institute for Animal Hygiene, Animal Welfare and Farm Animal Behavior, University of Veterinary Medicine Hannover (Foundation), 30173 Hannover, Germany; § EW GROUP GmbH, 49429 Visbek, Germany

**Keywords:** glucocorticoid, HPA axis, indicator, stress, domestic chicken

## Abstract

Studies indicate that the evaluation of animal welfare in birds may be carried out with the measurement of the stress-related hormone corticosterone in feathers. However a standardized procedure for corticosterone measurements in feathers is lacking, a validation needs to be carried out for each new species before implementation. The aim of the present study was to establish a valid method to measure corticosterone concentrations in feathers of laying hens in a precise and repeatable manner using an established and commercially available ELISA. Validation was performed with feather pools of tail and interscapular feathers of commercial Lohmann Brown laying hens. Assessment groups, consisting of 5 replicates, were created. All replicates of an assessment group were processed at the same time. Each replicate was run in 4 repetitions by ELISA. Intra-assay and interassay CV was 7.5 and 6.4%, respectively. The serial dilution showed linearity and parallelism. Examining the hormone extraction efficiency by using different methanol volumes resulted in no statistical differences (*P* > 0.05). Pulverized feathers showed higher corticosterone values than minced feathers (*P* > 0.05). Differences were shown between 2 feather types (tail vs. interscapular feathers; *P* < 0.05), as well as between vane and rachis (*P* < 0.05). Performance of a freeze–thaw cycle led to a decrease of corticosterone concentrations in the samples. A possible effect of UV-A radiation on the stability of corticosterone in the feathers was not found (*P* > 0.05). With the present study, a valid protocol, feasible for analyzing feather pools of laying hens, was developed. It may provide fundamentals for further investigations on corticosterone in feathers as a noninvasive indicator to evaluate aspects of animal welfare.

## Introduction

Animal welfare in livestock production has become increasingly important in recent years (Broom, 2010; Butterworth, 2013; Sandøe et al., 2020). As a consequence, animal welfare and especially the assessment of animal welfare is a focal point in various research fields (Mormède et al., 2007). There is consensus among different stakeholders and the academic landscape that animal welfare assessment heavily relies on the measurement and evaluation of environmental and animal-related signals (Mormède et al., 2007). In Germany, farmers bearing the responsibility for commercial livestock are legally obligated to evaluate the state of their animals using welfare-associated indicators (TierSchG, 2020). However, monitoring and evaluating animal welfare in farm animals needs to be feasible under commercial conditions and requires a competent and, at best, an objective and evidence-based view (Giersberg et al., 2017). In laying hens, the condition of the plumage and the integument acts as an indicator for feather pecking and cannibalism, both being behavioral disorders caused by various challenges the birds had or have to cope with (Sepeur et al., 2015; Giersberg et al., 2017). Recent studies showed that the evaluation of animal welfare in birds may be carried out objectively and noninvasively with the measurement of the stress-related hormone corticosterone in feathers (Bortolotti et al., 2008; Bortolotti et al., 2009; Fairhurst et al., 2011; Carbajal et al., 2014; Ganz et al., 2018; Johns et al., 2018; Weimer et al., 2018; Alba et al., 2019; von Eugen et al., 2019; Nordquist et al., 2020).

When exposed to certain stressors, the hypothalamic–pituitary–adrenal axis responds with the secretion of corticosterone in birds (Touma and Palme, 2005; Palme, 2019) and cortisol in most of the mammals (Palme, 2019). Consequently, the concentrations of corticosterone in the blood increase within min in captured wild birds of different species (Romero and Reed, 2005) as well as in laying hens (Beuving and Vonder, 1978) and decrease within h, depending on the initial stressor they have been exposed to (Beuving and Vonder, 1978). In humans, a half-life of circulating corticosterone of about 1 h is reported (Doggui, 2012); however, no values are found for birds. The quantification of hormone levels influenced by the hypothalamic–pituitary–adrenal axis has been applied over y as an indicator for stress and animal welfare in farm animals (Beuving and Vonder, 1978; Dehnhard et al., 2003; Rettenbacher et al., 2004; Odihambo Mumma et al., 2006; Mormède et al., 2007; Palme, 2019) and others (Bortolotti et al., 2008; Sheriff et al., 2011; Fairhurst et al., 2013; Schmaltz et al., 2016; Robertson et al., 2017; Peric et al., 2018; Palme, 2019). In addition to blood, several matrices, such as feces (Rettenbacher et al., 2004; Möstl et al., 2005; Touma and Palme, 2005; Weimer et al., 2018; Palme, 2019) and eggs (Rettenbacher et al., 2005; Schmaltz et al., 2016), are used to detect and quantify corticosterone or its metabolites in birds, whereas in other species, the use of saliva (Mormède et al., 2007), urine (Hay and Mormède, 1997), milk (Tucker and Schwalm, 1977), and hair (Arnon et al., 2016; Peric et al., 2018) is reported. Bortolotti et al. (2008) succeeded in detecting circulating corticosterone deposited in feathers of adult red-legged partridges (*Alectoris rufa*), which were exposed to stressors over wk during growth, when feathers are supplied with blood. Thereby, a promising tool was found, in contrast of measuring blood parameters, which react within a short period of time and therefore are less suitable to evaluate long-term liabilities (Mormède et al., 2007; Bortolotti et al., 2008), as we assume for poor animal welfare.

Subsequent studies on corticosterone in feathers were performed mostly in wild birds (e.g., Bortolotti et al., 2009; Koren et al., 2011; Lattin et al., 2011; Fairhurst et al., 2012; Lendvai et al., 2013; Harms et al., 2015; Kouwenberg et al., 2016; Aharon-Rotman et al., 2017; Freeman and Newman, 2018; Monclús et al., 2020), using feather corticosterone as a retrospective view on challenges the birds had to cope with during feather growth. Despite the wild birds, results of first investigations in poultry (Berkvens, 2012; Carbajal et al., 2014; Jenni-Eiermann et al., 2015; Zeinstra et al., 2015; Johns et al., 2017; Weimer et al., 2018; Alba et al., 2019; von Eugen et al., 2019; Nordquist et al., 2020; Lindenwald and Rautenschlein, unpublished data) are also encouraging; however, in the absence of a standardized procedure, authors applied different methods to detect and quantify corticosterone in feathers. These variations, such as variations in the amount of feather material or methanol volume for the extraction, crushed vs. grind up feathers, and different methods for filtration or different assays, make it rather impossible to compare the results, properly (Romero and Fairhurst, 2016).

As investigations on corticosterone in feathers are rather new in laying hens, a method validation is required, which includes the determination of precision, specificity, sensitivity, and accuracy (Buchanan and Goldsmith, 2004; Touma and Palme, 2005; Palme, 2019). This is essential, given that, to the best of our knowledge, no proper validation for commercial laying hens has been performed before and, especially, because noninvasive measurements of corticosterone and related hormones are finding their way into field studies, applied by researchers being new in the field of noninvasive endocrine assessments (Buchanan and Goldsmith, 2004). Alba et al. (2019) validated another method for domestic chickens, using a keratinase to digest the protein matrix in the first step. Berkvens (2012) validated a modified method for Barred Rock hens. Carbajal et al. (2014) evaluated a method for broilers. Thus, the objective of the present study was to establish a reliable and valid method to measure corticosterone concentrations in feathers of laying hens. Therefore, we focused on the assay validation and extraction efficiency first, using replicates, and thereafter, further technical influences (based on Bortolotti, 2010; Romero and Fairhurst, 2016) were examined, such as the manner of crushing the feathers (Newman and Freeman, 2018), different parts (Newman and Freeman, 2018) and types of feathers (Monclús et al., 2017; Weimer et al., 2018), as well as an effect of defrosting samples several times. Furthermore, as it was recommended by Romero and Fairhurst (2016), the effect of UV radiation on feather corticosterone stability was investigated briefly as hens are exposed to UV radiation in outdoor runs and also in floor husbandry systems where artificial light with a natural daylight spectrum is getting increasingly implemented because of animal welfare issues (Kämmerling et al., 2017; TierSchNutztV, 2017).

## Materials and methods

### Subjects

Generally, body feathers from the interscapular area (Carbajal et al., 2014; Monclús et al., 2017), hereinafter referred to as interscapular feathers (Monclús et al., 2017), and rectrices (Aharon-Rotman et al., 2017; Robertson et al., 2017; Freeman and Newman, 2018), hereinafter referred to as tail feathers (Aharon-Rotman et al., 2017; Robertson et al., 2017), were pulled from 11 adult laying hens. Feathers were collected from commercial Lohmann Brown laying hens (Lohmann Tierzucht GmbH, Cuxhaven, Germany), a commonly used genotype in Germany, as soon as discovering the bird's death. The animals originated from 7 flocks of commercial farms in Germany, where they were kept in accordance with local legislation (TierSchNutztV, 2017). Collected feathers were stored dark and dry in paper envelopes at room temperature as recommended by Bortolotti et al. (2009) and Monclús et al. (2017). Every feather was thoroughly cleaned (based on Jenni-Eiermann et al., 2014; von Eugen et al., 2019) with distilled water and degreased by bathing it in HPLC-grade methanol (Carl Roth GmbH + Co. KG, Karlsruhe, Germany) for 2 to 4 s (based on Robertson et al., 2017). Based on the studies by Lattin et al. (2011) and Freeman and Newman (2018), different feather pools were prepared, consisting of the same feather type of one animal or different animals, depending on the research question, as described in the chapters that follow, and subsequently processed. Table 1 gives an overview over the created pools.

**Table 1:** Feather pools created to corresponding research question.

Pools	N			
	Replicates	Feather type	Feathers	Laying hens
Interassay CV	2	Interscapular	25	1
Serial dilution	1	Interscapular	17	1
Methanol volume	25	Interscapular	38	1
Mincing	5	Tail	10	1
Pulverizing	5	Tail		
Tail	5	Tail		
Interscapular	5	Interscapular	17	
Vane	5	Tail	14	8
Rachis	5	Tail		
Freeze–thaw	3	Interscapular	25	1
UV-A radiation	10	Interscapular	80	3

### Corticosterone Extraction

Feather corticosterone extraction was undertaken using a modified procedure of that described by Bortolotti et al. (2008). In general, after removing the calamus of every feather, feathers of one pool (see Table 1) were crushed simultaneously, vortexed to homogenize the particles, and then aliquoted to samples of 10.0 mg (range of 9.5 mg to 10.5 mg; precision balance Mettler; Spoehrhase A.G., Giessen) each (based on Freeman and Newman, 2018). Up to 5 replicates were related to 1 assessment group, which went through the same treatment, depending on the research questions described as follows (see also Table 1). Replicates used for serial dilution and interassay variation, as well as the freeze–thaw cycle, amounted 50.0 mg (range of 49.5 mg to 50.5 mg) and 100.0 mg (range of 99.5 mg to 100.5 mg), respectively, for being expected to decrease in their levels. HPLC-grade methanol (Carl Roth GmbH + Co. KG) was added to each sample, and extraction was then initiated with an ultrasonic bath (VWR International, LLC, Radnor) for 30 min, followed by an incubation of 12 h (Freeman and Newman, 2018). Samples therefore were placed on a moving vortex platform at 50°C (Aharon-Rotman et al., 2017). Subsequently, feather particles of each sample were separated from methanol by pressure filtration using polyether sulfone syringe filters with a mesh diameter of 22 μm (Carl Roth GmbH + Co. KG). To avoid loss of extracted corticosterone, sample vials were washed twice using 1.0 mL of HPLC-grade methanol (Carl Roth GmbH + Co. KG) that was subsequently filtered and added to formerly filtered methanol from the sample. To evaporate the methanol, samples were placed into a water bath at 40°C until complete evaporation. Based on the studies by Harris et al. (2016), Harris et al. (2017), and Monclús et al. (2017) samples were resuspended in 500 μL of Tris-buffered saline, which was provided by the ELISA kit (Assay Buffer 15 by Enzo Life Sciences Inc., New York). Samples were frozen at −40°C for up to 12 h until examination; samples for long-term investigations, such as the interassay variation and the freeze–thaw cycle, were stored at −80°C.

### Assay Validation

The validation of the assay was performed in consideration of the recommendations by Buchanan and Goldsmith (2004), Sheriff et al. (2011), and Palme (2019). Feather corticosterone concentrations were analyzed using the commercial Enzo Life Sciences Corticosterone ELISA Kit ADI-901-097 (Enzo Life Sciences Inc.), a competitive immunoassay, also used by Bourgeon et al. (2014), Harris et al. (2016), and Harris et al. (2017), whereby samples were incubated with a sheep polyclonal antibody to corticosterone (Corticosterone ELISA Antibody by Enzo Life Sciences Inc.) over 2 h, first. After a washing procedure (Wash Buffer Concentrate by Enzo Life Sciences Inc.), a p-nitrophenyl phosphate (p-Npp Substrate by Enzo Life Sciences Inc.) was added, followed by a 1-h incubation. Finally, the Stop Solution (Enzo Life Sciences Inc.) completed the reaction.

Every sample was analyzed in 4 repetitions each. To validate the assay, all replicates related to the same research question were run in the same assay, with the exception of samples intended to calculate the interassay variation and the validation of the freeze–thaw cycle.

Precision of the ELISA was expressed via intra-assay and interassay CV. Intra-assay CV was calculated over all samples (n = 70 samples, each 4 repetitions). Interassay CV was examined by analyzing 2 replicates (each 4 repetitions) of an interscapular feather pool consisting of 25 feathers of 1 animal (Table 1). The 2 replicates were stored at −80°C and defrosted separately when analysis was performed.

Specificity of the ELISA was tested by examining the linearity of a serial dilution (Carbajal et al., 2014) and the parallelism of the serial dilution and the standard curve of each assay (Bourgeon et al., 2014; Carbajal et al., 2014; Glucs et al., 2018). Therefore, a replicate of a pool of interscapular feathers (17 feathers of 1 laying hen, Table 1) was used and diluted 1:2, 1:4, 1:5, and 1:10 with the assay buffer (Tris-buffered saline) before analyzing.

### Technical Issues

#### Extraction Efficiency

To examine the required quantity of methanol for a complete feather corticosterone extraction (Romero and Fairhurst, 2016), a pool of 38 interscapular feathers plucked from 1 laying hen was pulverized using a ball mill (MM-400; Retsch, Germany; also used by Ganz et al., 2018; see Table 1). Twenty-five replicates were created; of which, 5 were treated with 0.5 mL, 1.0 mL, 5.0 mL, 10.0 mL, or 15.0 mL HPLC-grade methanol (Carl Roth GmbH + Co. KG) each (based on the study by Newman and Freeman, 2018). Processing and analyzing of the replicates followed the procedure as described previously.

#### Mincing vs. Pulverizing

Investigations regarding the influence of the crushing method (Newman and Freeman, 2018) were performed using a pool of 10 tail feathers taken from 1 animal (see Table 1). All feathers were minced using scissors (following Bortolotti et al., 2008) and then vortexed. Half of the amount of the minced and vortexed feathers were further processed and pulverized using a ball mill (MM-400; Retsch, Germany; also used by Ganz et al., 2018). Therefore, the replicate was placed into a metal container, frozen in liquid nitrogen for 3 min to embrittle, and then pulverized for 1 min at 30 Hz. The minced and pulverized samples were divided into 5 replicates each and enriched with 5.0 mL HPLC-grade methanol (Carl Roth GmbH + Co. KG) to extract feather corticosterone as mentioned previously.

#### Tail vs. Interscapular Feathers

To investigate the effect of feather type (Monclús et al., 2017; Weimer et al., 2018), a pool of 10 tail feathers and a pool of 17 interscapular feathers of the same laying hen (Table 1) were pulverized. Five replicates each were taken, as described before. Extraction was performed using 5.0 mL of HPLC-grade methanol (Carl Roth GmbH + Co. KG) for each replicate.

#### Vane vs. Rachis

To examine potential differences of feather corticosterone concentrations within one feather, the rachis and vane (Newman and Freeman, 2018) of 14 tail feathers, distinguished and pooled from 8 different laying hens (Table 1), were analyzed. After separating the vane and rachis of feathers with a scalpel, the vane and rachis pools were pulverized separately and then aliquoted before feather corticosterone extraction was performed using 5.0 mL HPLC-grade methanol (Carl Roth GmbH + Co. KG) for each replicate.

#### Freeze–Thaw Cycle

A pool of 25 interscapular feathers of 1 animal was pulverized, and 3 replicates were created (Table 1) and extracted applying the aforementioned method. They were stored at −80°C. To examine the effect of the freeze–thaw cycle on feather corticosterone extraction and concentration, all replicates were defrosted 24 h after freezing, as part of the original protocol. While replicate 1 was analyzed after these 24 h, the remaining 2 replicates were frozen again and both defrosted after another 2 wk. While replicate 2 was then examined, the third replicate was frozen again until examination after another 16 wk. Therefore, the 3 replicates underwent a freeze–thaw cycle once, twice, or thrice and were frozen for 1, 15, and 113 D, respectively. All analyses were carried out as mentioned previously.

#### Effect of UV-A Radiation

A pool of 80 pulverized interscapular feathers, taken from 3 different laying hens (Table 1), was used to create 2 different groups, one for an UV-A treatment, and one as a control group. The material of each group was spread into a petri dish. Considering the total amount of radiation laying hens are exposed to with a lighting system for poultry during feather growth in the rearing period, the treatment group was placed 1 m beneath UV lights (LEDfactory B.V., Leeuwarden, the Netherlands) emitting a wavelength of 315 nm to 380 nm and a radiation power of 0.0676 Watt/m^2^ at room temperature for 18 D. The control group was placed in an opaque box and stored for 18 D beneath the treatment group. After 18 D, 5 replicates were created out of the groups and examined for feather corticosterone concentrations following the aforementioned procedure.

### Statistical Analyses

Calculation of feather corticosterone concentrations was performed as per the product manual of Enzo Life Sciences Corticosterone ELISA Kit ADI-901-097 (Enzo Life Sciences Inc.), whereby the standard curve fitting was performed using a 4-parameter logistic curve to interpolate feather corticosterone concentrations (also used by Gurung et al., 2018) by means of the Magellan data analysis software 7.2 (Tecan Group Ltd., Männedorf, Switzerland), after measurements of the optical density at 405 nm with an absorbance microplate reader (Tecan Group Ltd., Männedorf, Switzerland). Data management and calculations regarding descriptive statistics were performed using Microsoft Excel 2019 (Microsoft Corporation, Redmond). Statistical analyses were carried out using the software package Minitab 16.2.3 (Minitab LLC., State College). Feather corticosterone value of 1 sample was calculated as the arithmetic mean over the 4 repetitions. Generally, repetitions having a CV less than 20% were included in statistical analyses (based on Kinn Rød et al., 2017). Feather corticosterone values were converted from the unit pg/mL, given by the ELISA, to pg/mg, except for testing linearity and parallelism (based on Carbajal et al., 2014). Values of the diluted samples were plotted against the calculated corticosterone concentrations (Carbajal et al., 2014). For the parallelism test, results were logarithmized to the base 10, and a linear regression was calculated (based on Carbajal et al., 2014). To assess distribution, Anderson–Darling normality test was calculated. To show possible differences, a Kruskal–Wallis test was performed for the methanol groups. With the Mann–Whitney U-test, differences between the other groups (mincing vs. pulverizing, tail vs. interscapular feathers, vane vs. rachis, effect of UV-A radiation) were tested. Significance was assumed at the level of *P* < 0.05.

## Results

### Assay Validation

**Figure 1:**
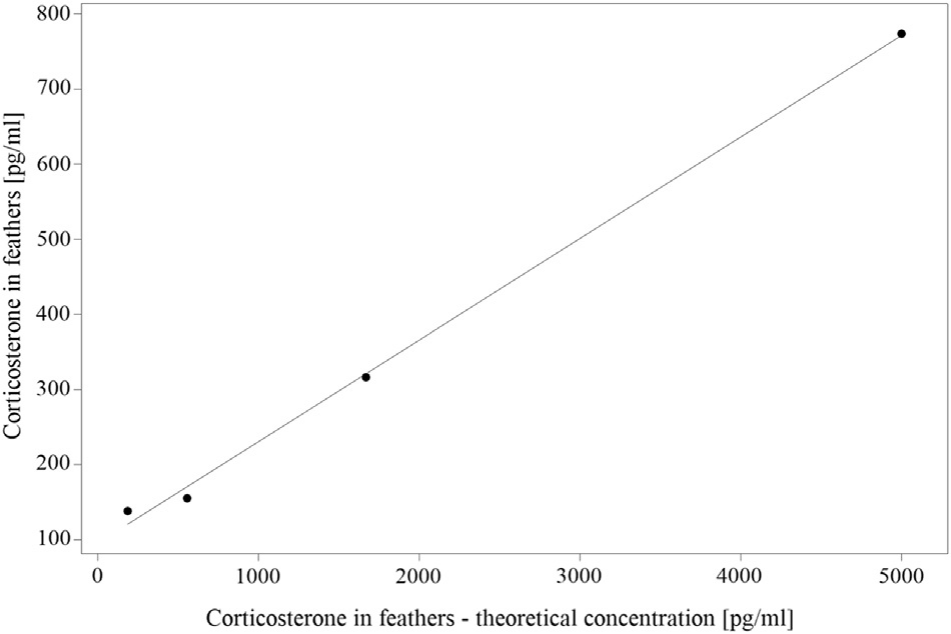
Linearity of the serial dilution.

**Figure 2:**
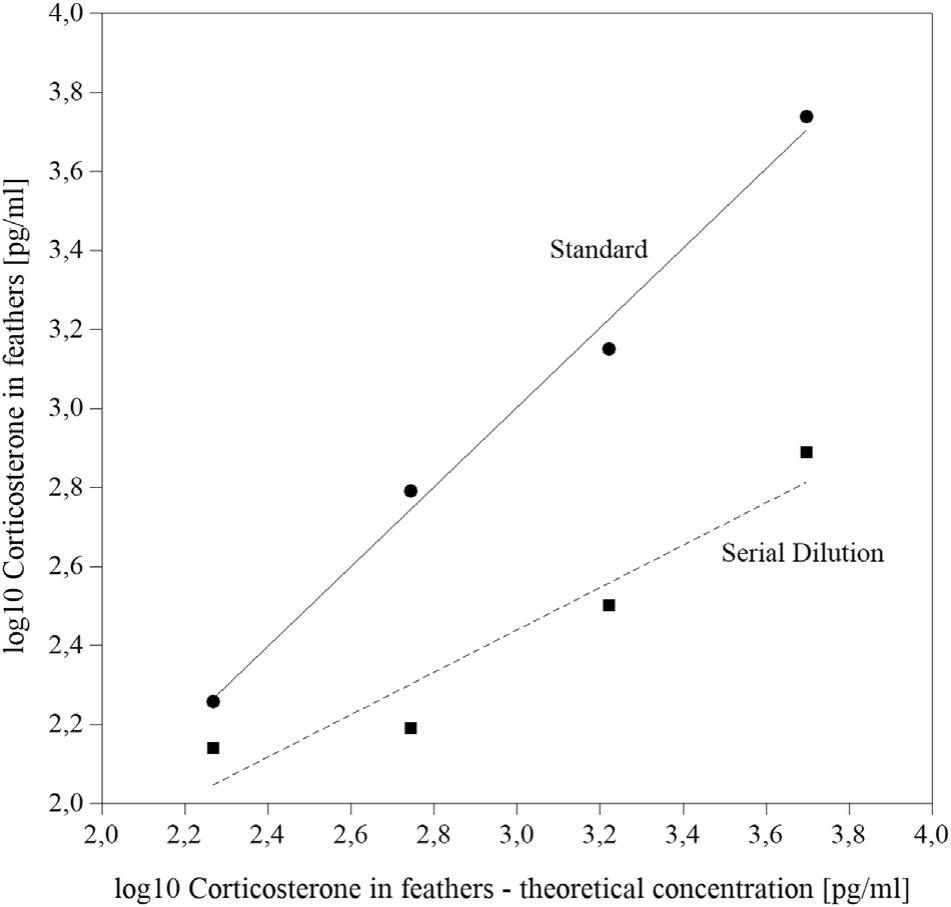
Parallelism test of the serial dilution and the standard curve.

Intra-assay CV over all samples was in average 7.5% (median, n = 70 samples), whereas interassay CV was 6.4% (n = 2 samples). Linearity of the serial dilution (n = 4 diluted samples) was R^2^_linearity_ = 0.997, described by the formula y_linearity_ = 0.1352x + 95.58 (Figure 1). Parallelism of the serial dilution and the standard curve is shown in Figure 2, with R^2^_dilution_ = 0.873 (y_dilution_ = 0.5358x + 0.8315) and R^2^_standard_ = 0.989 (y_standard_ = 1.103x – 119.3), respectively.

### Technical Issues

#### Extraction Efficiency

**Figure 3:**
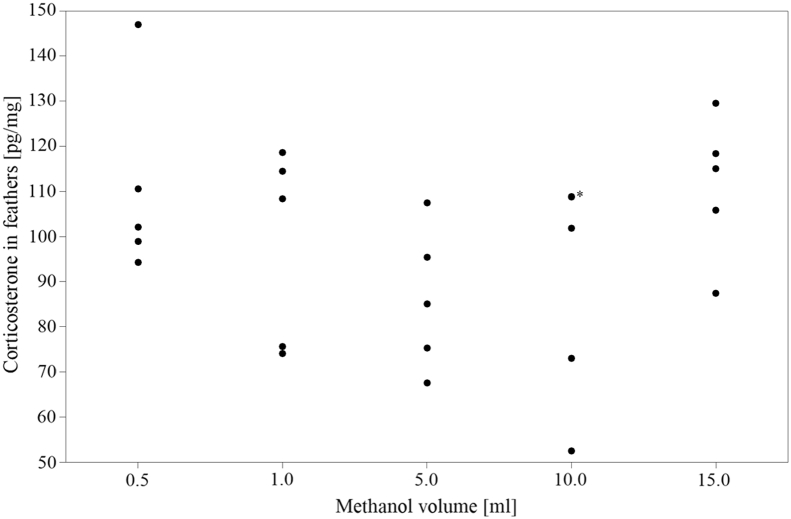
Effect of different methanol volumes (n = 5 replicates each group; ∗: 2 values, 108.9 pg/mg and 108.8 pg/mg, are overlapping).

Varying the methanol volume for feather corticosterone extraction did not show any significant differences (*P* = 0.204; Figure 3).

#### Mincing vs. Pulverizing

Although not being significant, feather corticosterone concentrations of the samples being crushed by scissors resulted in lower values (19.3 pg/mg, SD 5.00 pg/mg, n = 5) than those of samples treated with the ball mill (23.3 pg/mg, SD 3.13 pg/mg, n = 5; *P* = 0.296; see also Table 2).

**Table 2:** Feather corticosterone concentrations of different pools in pg/mg, each pool consisting of n = 5 replicates.

Pools	Mean	SD	Median	Minimum	Maximum
Tail feathers (minced)	19.3	5.00	22.5	11.1	24.3
Tail feathers (pulverized)	23.3	3.13	24.2	18.2	26.8
Interscapular feathers	80.0	18.14	79.5	48.8	92.2
Vane	61.7	15.06	54.9	43.5	87.2
Rachis	23.3	3.43	25.4	19.1	27.8
UV-A group	49.4	10.51	44.0	37.9	67.0
Control group	42.6	10.58	40.2	32.1	61.2

#### Tail vs. Interscapular Feathers

Feather corticosterone concentrations in tail feathers were significantly (*P* = 0.012) lower (23.3 pg/mg, SD 3.13 pg/mg, n = 5) than those of interscapular feathers (80.0 pg/mg, SD 18.14 pg/mg, n = 5; Table 2).

#### Vane vs. Rachis

Feather corticosterone concentrations of the vane and rachis were assessed separately and showed significant differences (*P* = 0.012) with 61.7 pg/mg (SD 15.06 pg/mg, n = 5) in the vanes and 23.3 pg/mg (SD 3.43 pg/mg, n = 5) in the rachises (Table 2).

#### Freeze–Thaw Cycle

The initial feather corticosterone concentration of the measurement was 25.3 pg/mg (SD 1.05 pg/mg, 4 repetitions). The concentration decreased within the cycle, amounting 17.2 pg/mg (SD 1.25 pg/mg, 4 repetitions) after defrosting twice, up to a final value of 8.0 pg/mg (SD 0.56 pg/mg, 4 repetitions) after defrosting thrice.

#### Effect of UV-A Radiation

Exposure to UV-A radiation did not affect concentrations or traceability of corticosterone in feathers (*P* = 0.403). Mean feather corticosterone concentration of the UV-A–treated samples was 49.4 pg/mg (SD 10.51 pg/mg, n = 5), whereas the control group samples had a mean of 42.6 pg/mg (SD 10.58 pg/mg, n = 5; Table 2).

## Discussion

Measuring corticosterone in feathers of laying hens may be a suitable tool to evaluate birds’ welfare. Moreover, feather corticosterone measurements may be valuable for an indicator-based flock management as flocks and individuals, who had to cope with adverse husbandry conditions during feather growth in the rearing period and are therefore susceptible to develop behavioral disorders (e.g., feather pecking and cannibalism), can be identified and treated accordingly. In addition, a correlation between altered feather corticosterone levels and behavioral disorders of individual birds would enable breeders to implement corticosterone in feathers in breeding schemes to provide stress resilient genetics, to address the occurrence of feather pecking, which is a heritable trait but difficult to quantify directly (Grams et al., 2015). The present study suggests a reliable protocol for measuring corticosterone in feathers; however, there are still unclear aspects when quantifying it, such as the deposition of corticosterone into feathers (Bortolotti et al., 2010) per se. Jenni-Eiermann et al. (2015) addressed this issue in their research with feathers of pigeons and were able to recover injected and labeled corticosterone. Additional “unresolved technical issues” (Romero and Fairhurst, 2016), such as influences on the deposition (Romero and Fairhurst, 2016) and the so-called small sample artifact (Lattin et al., 2011; Berk et al., 2016), which describes the appearance of higher feather corticosterone concentrations in small sample masses, compared with larger ones, are discussed. Therefore, besides studies on effects of stressors influencing feather corticosterone levels, different validation studies have already been performed in different species (Lattin et al., 2011; Carbajal et al., 2014; Berk et al., 2016; Harris et al., 2016; Robertson et al., 2017; Freeman and Newman, 2018). Furthermore, an official abbreviation for corticosterone should be established (Raff, 2016), such as **ACTH** for adrenocorticotropic hormone.

### Assay Validation

The present study performed an analytical validation, through the determination of precision, specificity, sensitivity, and accuracy (Palme, 2019) and the investigation on few technical issues. Yet, a physiological or biological validation to fulfill a complete validation as recommended by Palme (2019) is missing, owing to the lack of a suitable method (Berk et al., 2016): Studies analyzing corticosterone concentrations in serum, plasma, or excreta commonly use an ACTH challenge test for physiological validation (Palme, 2019). In domestic chickens, several studies using ACTH are reported (Dehnhard et al., 2003; Rettenbacher et al., 2004; Touma and Palme, 2005; Odihambo Mumma et al., 2006), showing a responsive hypothalamic–pituitary–adrenal axis. However, the ACTH challenge test is based on reactions within h; thus, a method to perform an adequate physiological validation of corticosterone in feathers growing over wk is desired (Berk et al., 2016).

Results of intra-assay and interassay CV being lower than 10%, as calculated in the present study, indicate a good precision of the assay (Carbajal et al., 2014). Using the same ELISA kit as in the present study, Bourgeon et al. (2014), Harris et al. (2016), and Harris et al. (2017) achieved comparable results. In addition, a more precise assessment could be done using low and high concentrated feather corticosterone samples (Palme, 2019). Yet, the present values were in the range of what can be achieved for intra-assay and interassay CV as per the product manual for the assay (Enzo Life Sciences Inc., 2019). The linearity of the serial dilution indicated a good specificity, also shown in broilers by Carbajal et al. (2014). Furthermore, it showed that measured feather corticosterone concentrations are in the quantitative range of the assay. The parallelism test led to acceptable results: R^2^ for the standard curve was comparable with that of the study by Carbajal et al. (2014), who achieved 0.988. However, their modified standard curve showed a higher R^2^ than that of the present study (0.934 vs. 0.873). Serial dilutions not being parallel with the standard curve may be affected from interfering substances (Bourgeon et al., 2014; Freeman and Newman, 2018). Reference values regarding cross reactivity and sensitivity of the assay were taken from the product manual of the ELISA kit (Arnon et al., 2016), mentioning 28.6% for deoxycorticosterone, 1.7% for progesterone, and several derivatives of cholesterol having a cross reactivity lower than 0.28%, and the lowest detection limit for corticosterone is represented at 26.99 pg/mL (Enzo Life Sciences Inc., 2019). To the best of our knowledge, no recombinant deoxycorticosterone of chickens is available to check the cross reactivity for them. Cross reactivity is only described for unsaturated steroids but not for 5α- or 5β-reduced corticosterone metabolites (Enzo Life Sciences Inc., 2019). This has to be considered when interpreting results. On the other hand, the slope of the serial dilutions being smaller than the slope of the standard curve (Figure 2) may indicate that, instead of unspecific binding, there may be less binding of actually available corticosterone. This can be explainable by the results of Kinn Rød et al. (2017) finding less corticosterone in the Enzo Life Sciences ELISA Kit compared with others. The affinity for chickens' corticosterone coming from feathers may be lower in some binding sites of the polyclonal antibody. Considering parallelism, an inappropriate sample mass should be taken into account, which emphasizes the importance of a consistent sample mass. Freeman and Newman (2018) determined the optimal sample mass for feathers of the wild turkey (*Meleagris gallopavo*), Canada jay (*Perisoreus canadensis*), and black-capped chickadee (*Poecile atricapillus*) by serial dilutions of different sample masses. Applying this in further investigations on corticosterone in feathers of laying hens may improve the present procedure. Regarding accuracy, spike recovery by the application of the Enzo Life Sciences Corticosterone ELISA kit was assessed by Aharon-Rotman et al. (2017) using plasma samples of house sparrows (*Passer domesticus*), spiked with tritiated corticosterone. They achieved an average accuracy of 92.2 ± 2.1% (Aharon-Rotman et al., 2017). Spike recovery for this kit using feathers of chickens is lacking.

Unlike Bortolotti et al. (2008) who recommend the unit pg/mm for feather corticosterone concentrations, samples used in the present study were standardized on mass, using pg/mg (Monclús et al., 2017; Robertson et al., 2017; Freeman and Newman, 2018). This seems to be reasonable for the authors as the aim was to compare the same sample under different treatments and to determine their repeatability, rather than investigate feather corticosterone concentrations within a single feather under consideration of its growth rate. Consequently, replicates were created of feather pools (Lattin et al., 2011; Freeman and Newman, 2018). Applying this methodology in the present study avoided the small sample artifact as every sample had the same weight (Lattin et al., 2011; Berk et al., 2016). On the other hand, weighing variations may also lead to high variance in results (Bortolotti, 2010), which has to be taken into account when applying this method. In addition, it should be considered that comparing absolute values between studies based on modified procedures could lead to incorrect conclusions (Palme, 2019). Investigations on different commercial ELISA kits showed that comparisons can be carried out based on relative values at most (Abelson et al., 2016; Kinn Rød et al., 2017) but not on “true values” (Kinn Rød et al., 2017). An external standard for analyzing corticosterone in feathers via an ELISA is not known. These results emphasize the need for researchers to evaluate the procedure they use in their own standardized way (Berk et al., 2016) and to describe and point out modifications as long as an official standardization is lacking. Nevertheless, the range of corticosterone concentrations in broiler feathers measured by Carbajal et al. (2014) via an ELISA in ng/mL is comparable with the values given in pg/mL from the ELISA used in the present study. This underlines the necessity of investigations on species-specific values (Fairhurst et al., 2012, 2013; Kouwenberg et al., 2016) or a species-specific curve of sample mass vs. corticosterone in feathers (Lattin et al., 2011). Other aspects to be considered when evaluating and comparing results are birds’ genotype and age: Jenni-Eiermann et al. (2015) showed that the deposition of corticosterone into feathers may also be affected by melanism, which has to be taken into account when comparing results from white and brown layers, respectively. Monclús et al. (2017) did not find different values in birds of different ages.

### Technical Issues on Corticosterone Extraction

#### Extraction Efficiency

An appropriate validation requires an efficient hormone extraction (Buchanan and Goldsmith, 2004). In the present study, 5 different volumes of methanol were used for extraction purposes; however, no differences in feather corticosterone values were found, which is in accordance with the study by Freeman and Newman (2018), using 5.0 mL and 10.0 mL of methanol (*P* > 0.05), respectively. Thus, we conclude, a saturation of methanol was not achieved, and corticosterone was extracted completely from the feathers. The decision of using 5.0 mL for the further group treatments was based on the fact that this volume showed the smallest variation of feather corticosterone values (see Figure 3) combined with practical issues, such as handling the samples and the duration of evaporation.

#### Mincing vs. Pulverizing

Freeman and Newman (2018) found higher feather corticosterone concentrations when feathers were pulverized than those when feathers being minced by scissors (*P* < 0.05). Although no significant difference between the groups was found in the present study, results show the same tendency (*P* > 0.05). Pulverization increases the surface of the sample and thus may explain the increased feather corticosterone values (Sheriff et al., 2011; Romero and Fairhurst, 2016; Freeman and Newman, 2018). In contrast to Freeman and Newman (2018), results of the minced samples showed a higher variability in the present study, which may simply be explained with the lack of homogeny when compared with pulverized samples. Consequently, the ball mill treatment was applied for the other groups in this study.

#### Tail vs. Interscapular Feathers

As expected, different feather types of the same bird showed significant differences in feather corticosterone concentrations (*P* < 0.05). Different feather types grow during different periods of time with different growth rates (Rohwer and Rohwer, 2013; Häffelin and Andersson, unpublished data) and thus are exposed to corticosterone over different durations (Monclús et al., 2017). In addition, the difference in structure between feather types may have an impact on the deposition of corticosterone into feathers (Romero and Fairhurst, 2016). Monclús et al. (2017) did not find a correlation between primary and interscapular feathers. Weimer et al. (2018) found “a strong correlation” between corticosterone in primary feathers and body feathers from the interscapular area, grown at the same time, administering synthetic corticosterone to broilers via the drinking water for 72 h. Surprisingly, elevated feather corticosterone concentrations could be measured beginning 6 h after application (Weimer et al., 2018), which is hardly comprehensible when considering growth rates of feathers to be around wk (Serra and Underhill, 2006; Butler et al., 2008; Oschadleus and Underhill, 2008; Rohwer and Rohwer, 2013; Häffelin and Andersson, unpublished data). Consequently and based on the studies by Romero and Fairhurst (2016) and Monclús et al. (2017), it is recommended to use the same type of feather when analyzing and comparing feather corticosterone concentrations as the feathers have the same structure and time of growth. When drawing comparisons, feathers should origin from the same replacement generation as Monclús et al. (2020) found different levels of feather corticosterone in the same bird but from different seasons. Concerning the most suitable feather type for feather corticosterone analysis, very small feathers, such as body feathers from the belly and the flanks, were ruled out, as they were broken frequently. Previous investigations were also performed using the same feather types as in the present study (Carbajal et al., 2014; Aharon-Rotman et al., 2017; Monclús et al., 2017; Robertson et al., 2017; Freeman and Newman, 2018).

#### Vane vs. Rachis

Showing higher feather corticosterone levels in the vane than in the rachis of tail feathers is in accordance with the findings of Freeman and Newman (2018; *P* < 0.05). Owing to the fact that the total weight of a feather is mainly made up of the rachis, one must be aware of choosing feathers not only of the same feather type but with the same weight. Another possibility is to refer the feather corticosterone concentration on feather length in pg/mm (Bortolotti et al., 2008; Romero and Fairhurst, 2016).

#### Freeze–Thaw Cycle

All samples of the present study were frozen until examination (Bortolotti et al., 2008). This procedure was mainly performed because of practical reasons and is however a fixed part of the protocol. Studies on freeze–thaw cycles using corticosterone originated for example from mouse serum (Kang et al., 2013) or mouse plasma (Li et al., 2015) showed that levels do not alter. However, comparisons of the values measured in the present study after freeze–thaw cycles showed decrease of feather corticosterone concentrations up to a third from the initial value. The buffer coming from the ELISA kit used in the present study, and in which the hormone was stored frozen, may not be an adequate matrix to freeze and thaw feather corticosterone samples. Thus, researchers should avoid freezing samples that have been defrosted, when planning their experimental design.

#### Effect of UV Radiation

Samples being exposed to UV-A radiation did not differ from the control group samples regarding feather corticosterone concentrations. However, the effect of UV radiation remains to be studied in detail for free range hens as natural UV radiation percentage varies during seasons, time of day, and location (Kämmerling et al., 2017) and may have an effect on corticosterone deposition during growth of feathers. Nevertheless, the current results indicate that the stability of corticosterone already deposited into feathers is not influenced by UV-A radiation. This finding allows to compare results of feathers of hens being exposed to UV-A light through the lighting system with hens that were not exposed to UV-A radiation. However, the aforementioned effects of light parameters remain to be investigated.

## Conclusion

The aim of the present study was to establish a reliable and valid method to measure corticosterone concentrations in feathers of laying hens. The presented results indicated that the applied technique and methodology, and thus the extraction procedure and assay kit, qualifies as valid. In this study, the groundwork for future investigations of reference values for laying hens was laid. To draw comparisons and gain information about response of birds and flocks to and in different environmental conditions, the use of the same extraction procedure and assay kit proposed in the present study is essential. A possible correlation between corticosterone concentrations in feathers and in the blood should be clarified. Further investigations should be performed on whether feather corticosterone is suitable as an indicator for animal welfare in laying hens. With additionally obtained information on those issues, the present method may have potential for an evidence-based assessment of animal welfare in laying hens, which can be applied noninvasively. As corticosterone in feathers is related to animal welfare it may also be suitable to assess and enhance husbandry conditions and production systems regarding animal welfare. Moreover, corticosterone in feathers may also be implemented in breeding schemes to provide stress resilient strains (genotype × environment interaction). Samples from individual marked and pedigreed birds need to be analyzed for estimating genetic parameters for feather corticosterone values at different ages and management conditions. In the last decade, examinations of corticosterone in feathers have been performed basically in wild birds, yet the potential has not been exhausted for commercial poultry.

## References

[bib1] Abelson K.S.P., Kalliokoski O., Teilmann A.C., Hau J. (2016). Applicability of commercially available ELISA kits for the quantification of faecal immunoreactive corticosterone metabolites in mice. In Vivo.

[bib2] Ag Guide (2010). Guide for the Care and Use of Agricultural Animals in Research and Teaching (Ag Guide).

[bib3] Aharon-Rotman Y., Buchanan K.L., Klaasen M., Buttemer W.A. (2017). An experimental examination of interindividual variation in feather corticosterone content in the house sparrow, *Passer domesticus* in southeast Australia. Gen. Comp. Endocrinol..

[bib4] Alba A.C., Strauch T.A., Keisler D.H., Wells K.D., Kesler D.C. (2019). Using a keratinase to degrade chicken feathers for improved extraction of glucocorticoids. Gen. Comp. Endocrinol..

[bib5] Arnon L., Hazut N., Tabachnik T., Weller A., Koren L. (2016). Maternal testosterone and reproductive outcome in a rat model of obesity. Theriogenology.

[bib6] Berk S.A., McGettrick J.R., Hansen W., Breuner C. (2016). Methodological considerations for measuring glucocorticoid metabolites in feathers. Conserv. Physiol..

[bib7] Berkvens C.N. (2012). Keratin Glucocorticoid Analysis by Enzyme Immunoassay in Mammals, Birds and Reptiles.

[bib8] Beuving G., Vonder G.M.A. (1978). Effect of stressing factors on corticosterone levels in the plasma of laying hens. Gen. Comp. Endocrinol..

[bib9] Bortolotti G.R. (2010). Flaws and pitfalls in the chemical analysis of feathers: bad news–good news for avian chemoecology and toxicology. Ecol. Appl..

[bib10] Bortolotti G.R., Marchant T.A., Blas J., Cabezas S. (2009). Tracking stress: localisation, deposition and stability of corticosterone in feathers. J. Exp. Biol..

[bib11] Bortolotti G.R., Marchant T.A., Blas J., German T. (2008). Corticosterone in feathers is a long-term, integrated measure of avian stress physiology. Funct. Ecol..

[bib12] Bourgeon S., Leat E.H.K., Magnusdóttir E., Furness R.W., Strøm H., Petersen A., Gabrielsen G.W., Hanssen S.A., Bustnes J.O. (2014). Feather corticosterone levels on wintering grounds have no carry-over effects on breeding among three populations of great skuas (*Stercorarius skua*). PloS ONE.

[bib13] Broom D.M. (2010). Animal welfare: an aspect of care, sustainability, and food auality required by the public. J. Vet. Med. Educ..

[bib14] Buchanan K.L., Goldsmith A.R. (2004). Noninvasive endocrine data for behavioural studies: the importance of validation. Anim. Behav..

[bib15] Butler L.K., Hayden T.J., Romero L.M. (2008). Prebasic molt of black-capped and white-eyed Vireos: effects of breeding site and the el Niño-Southern oscillation. Condor.

[bib16] Butterworth A. (2013). On-farm broiler welfare assessment and associated training. Rev. Bras. Cienc. Avic..

[bib17] Carbajal A., Tallo-Parra O., Sabes-Alsina M., Mular I., Lopez-Bejar M. (2014). Feather corticosterone evaluated by ELISA in broilers: a potential tool to evaluate broiler welfare. Poult. Sci..

[bib18] Dehnhard M., Schreer A., Krone O., Jewgenow K., Krause M., Grossmann R. (2003). Measurement of plasma corticosterone and fecal glucocorticoid metabolites in the chicken (*Gallus domesticus*), the great cormorant (*Phalacrocorax carbo*), and the goshawk (*Accipiter gentilis*). Gen. Comp. Endocrinol..

[bib19] Doggui R. (2012). Immunoanalytical characteristics of corticosterone. Immuno-analyse et biologie spécialisée.

[bib20] Enzo Life Sciences Inc (2019). Product manual, corticosterone ELISA kit, ADI-901-097. https://www.enzolifesciences.com/fileadmin/files/manual/ADI-901-097_insert.pdf.

[bib21] Fairhurst G.D., Frey M.D., Reichert J.F., Szelest I., Kelly D.M., Bortolotti G.R. (2011). Does environmental enrichment reduce stress? An integrated measure of corticosterone from feathers provides a novel perspective. PloS ONE.

[bib22] Fairhurst G.D., Marchant T.A., Soos C., Machin K.L., Clark R.G. (2013). Experimental relationship between levels of corticosterone in plasma and feathers in a free-living bird. J. Exp. Biol..

[bib23] Fairhurst G.D., Navarro J., González-Solis J., Marchant T.A., Bortolotti G.R. (2012). Feather corticosterone of a nestling seabird reveals consequences of sex-specific parental investment. Proc. Biol. Sci..

[bib24] Freeman N.E., Newman A.E.M. (2018). Quantifying corticosterone in feathers: validations for an emerging technique. Conserv. Physiol..

[bib25] Ganz K., Jenny D., Kraemer T., Jenni L., Jenni-Eiermann S. (2018). Prospects and pitfalls of using feathers as a temporal archive of stress events and environmental pollutants: a review and case study. J. Ornithol..

[bib26] Giersberg M.F., Spindler B., Kemper N. (2017). Assessment of plumage and integument condition in dual-purpose breeds and conventional layers. Animals.

[bib27] Glucs Z.E., Smith D.R., Tubbs C.W., Jones Scherbinski J., Welch A., Burnett J., Clark M., Eng C., Finkelstein M.E. (2018). Glucocorticoid measurement in plasma, urates, and feathers from California condors (*Gymnogyps californianus*) in response to a human-induced stressor. PLoS ONE.

[bib28] Grams V., Bögelein S., Grashorn M.A., Bessei W., Bennewitz J. (2015). Quantitative genetic analysis of traits related to fear and feather pecking in laying hens. Behav. Genet..

[bib29] Gurung S., Hoffman J., Stringfellow K., Abi-Ghanem D., Zhao D., Caldwell D., Lee J., Styles D., Berghman L., Byrd J., Farnell Y., Archer G., Farnell M. (2018). Depopulation of caged layer hens with a compressed air foam system. Animals.

[bib30] Harms N.J., Legagneux P., Gilchrist H.G., Bêty J., Love O.P., Forbes M.R., Bortolotti G.R., Soos C. (2015). Feather corticosterone reveals effect of moulting conditions in the autumn on subsequent reproductive output and survival in an Arctic migratory bird. Proc. R. Soc. B..

[bib31] Harris C.M., Madliger C.L., Love O.P. (2016). Temporal overlap and repeatability of feather corticosterone levels: practical considerations for use as a biomarker. Conserv. Physiol..

[bib32] Harris C.M., Madliger C.L., Love O.P. (2017). An evaluation of feather corticosterone as a biomarker of fitness and an ecologically relevant stressor during breeding in the wild. Oecologia.

[bib33] Hay M., Morméde P. (1997). Determination of catecholamines and methoxycatecholamines excretion patterns in pig and rat urine by ion-exchange liquid chromatography with electrochemical detection. J. Chromatogr. B.

[bib34] Jenni-Eiermann S., Helfenstein F., Vallat A., Glauser G., Jenni L. (2015). Corticosterone: effects on feather quality and deposition into feathers. Methods Ecol. Evol..

[bib35] Johns D.W., Marchant T.A., Fairhurst G.D., Speakman J.R., Clark R.G. (2018). Biomarker of burden: feather corticosterone reflects energetic expenditure and allostatic overload in captive waterfowl. Funct. Ecol..

[bib36] Kämmerling D.J., Döhring S., Arndt C., Andersson R. (2017). Daylight in barn – spectrum specification for light sources in poultry. Berl. Münch. Tierärztl. Wochenschr..

[bib37] Kang L., Jiang T., Ge X., Peng L., Xie Y., Luan X., Li H., Rong Z., Qi H., Chen H. (2013). Determination of the stress biomarker corticosterone in serum of tumor-bearing mice by surrogate-based liquid chromatography-tandem mass spectrometry. Biomed. Chromatogr..

[bib38] Kinn Rød A.M., Harkestad N., Jellestad F.K., Murison R. (2017). Comparison of commercial ELISA assays for quantification of corticosterone in serum. Scientific Rep..

[bib39] Koren L., Nakagawa S., Burke T., Soma K.K., Wynne-Edwards K.E., Geffen E. (2011). Non-breeding feather concentrations of testosterone, corticosterone and cortisol are associated with subsequent survival in wild house sparrows. Proc. Biol. Sci..

[bib40] Kouwenberg A., Hipfner J.M., McKay D.W., Storey A.E. (2016). Corticosterone levels in feathers and blood of rhinoceros auklets *Cerorhinca monocerata* are affected by variation in environmental conditions. Mar. Biol..

[bib41] Lattin C.R., Reed J.M., DesRochers D.W., Romero L.M. (2011). Elevated corticosterone in feathers correlates with corticosterone-induced decreased feather quality: a validation study. J. Avian Biol..

[bib42] Lendvai Á.Z., Giraudeau M., Németh J., Bakó V., McGraw K.J. (2013). Carotenoid-based plumage coloration reflects feather corticosterone levels in male house finches (*Haemorhous mexicanus*). Behav. Ecol. Sociobiol..

[bib43] Li H., Sheng L.-P., Wang B., Yang Z.-L., Liu S.-Y. (2015). An optimized method for corticosterone analysis in mouse plasma by ultra-performance liquid chromatography-full-scan high resolution accurate mass spectrometry. J. Chromatogr. Sci..

[bib44] Monclús L., Carbajal A., Tallo-Parra O., Sabés-Alsina M., Darwich L., Molina-López R.A., Lopez-Bejar M. (2017). Relationship between feather corticosterone and subsequent health status and survival in wild Eurasian Sparrowhawk. J. Ornithol..

[bib45] Monclús L., Tallo-Parra O., Carbajal A., Quevedo M.A., Lopez-Bejar M. (2020). Feather corticosterone in Northern Bald Ibis *Geronticus eremita*: a stable matrix over time able to predict reproductive success. J. Ornithol..

[bib46] Mormède P., Andanson S., Aupérin B., Beerda B., Guémené D., Malmkvist J., Manteca X., Manteuffel G., Prunet P., van Reenen C.G., Richard S., Veissier I. (2007). Exploration of the hypothalamic–pituitary–adrenal function as a tool to evaluate animal welfare. Physiol. Behav..

[bib47] Möstl E., Rettenbacher S., Palme R. (2005). Measurement of corticosterone metabolites in birds' droppings: an analytical approach. Ann. N. Y. Acad. Sci..

[bib48] Nordquist R.E., Zeinstra E.C., Dougherty A., Riber A.B. (2020). Effects of dark brooder rearing and age on hypothalamic vasotocin and feather corticosterone levels in laying hens. Front. Vet. Sci..

[bib49] Odihambo Mumma J., Thaxton J.P., Vizzier-Thaxton Y., Dodson W.L. (2006). Physiological stress in laying hens. Poult. Sci..

[bib50] Oschadleus H.D., Underhill L.G. (2008). Primary moult of adult Red-billed Queleas (*Quelea quelea*) in southern Africa in relation to patterns of movement. Emu.

[bib51] Palme R. (2019). Non-invasive measurement of glucocorticoids: Advances and problems. Physiol. Behav..

[bib52] Peric T., Comin A., Corazzin M., Montillo M., Canavese F., Stebel M., Prandi A. (2018). Hair cortisol concentrations in New Zealand white rabbits subjected to surgery. Anim. Welfare.

[bib53] Raff H. (2016). CORT, Cort, B, corticosterone, and now cortistatin: enough already!. Endocrinology.

[bib54] Rettenbacher S., Möstl E., Hackl R., Ghareeb K., Palme R. (2004). Measurement of corticosterone metabolites in chicken droppings. Br. Poult. Sci..

[bib55] Rettenbacher S., Möstl E., Hackl R., Palme R. (2005). Corticosterone in chicken eggs. Ann. N. Y. Acad. Sci..

[bib56] Robertson J.K., Muir C., Hurd C.S., Hing J.S., Quinn J.S. (2017). The effect of social group size on feather corticosterone in the co-operatively breeding Smooth-billed Ani (*Crotophaga ani*): an assay validation and analysis of extreme social living. PLoS ONE.

[bib57] Rohwer V.G., Rohwer S. (2013). How do birds adjust the time required to replace their flight feathers?. The Auk.

[bib58] Romero L.M., Fairhurst G.D. (2016). Measuring corticosterone in feathers: Strength, limitations, and suggestions for the future. Comp. Biochem. Physiol. A. Physiol..

[bib59] Romero L.M., Reed J.M. (2005). Collecting baseline corticosterone samples in the field: is under 3 min good enough?. Comp. Biochem. Physiol. A. Physiol..

[bib60] Sandøe P., Hansen H.O., Rhode H.L.H., Houe H., Palmer C., Forkman B., Christensen T. (2020). Benchmarking Farm Animal Welfare – a novel tool for cross-country comparison applied to pig production and pork consumption. Animals.

[bib61] Schmaltz G., Quinn J.S., Schoech S.J. (2016). Maternal corticosterone deposition in avian yolk: influence of laying order and group size in a joint-nesting, cooperatively breeding species. Gen. Comp. Endocrinol..

[bib62] Sepeur S., Spindler B., Schulze-Bisping M., Habig C., Andersson R., Beyerbach M., Kemper N. (2015). Comparison of plumage condition of laying hens with intact and trimmed beaks kept on commercial farms. Europ. Poult. Sci..

[bib63] Serra L., Underhill L.G. (2006). The regulation of primary molt speed in the Grey Plover, *Pluvialis squatarola*. Acta Zoologica Sinica.

[bib64] Sheriff M.J., Dantzer B., Delehanty B., Palme R., Boonstra R. (2011). Measuring stress in wildlife: techniques for quantifying glucocorticoids. Oecologia.

[bib65] TierSchNutztV (2017). German Legal Standard on the Protection of Animals and Animal Husbandry Conditions. Tierschutz-Nutztierhaltungsverordnung in der Fassung der Bekanntmachung vom 22. August 2006 (BGBl. I S. 2043), die durch Artikel 3 Absatz 2 des Gesetzes vom 30. Juni 2017 (BGBl. I S. 2147) geändert worden ist. https://www.gesetze-im-internet.de/tierschnutztv/TierSchNutztV.pdf.

[bib66] TierSchG (2020). German Animal Welfare Act. Tierschutzgesetz in der Fassung der Bekanntmachung vom 18. Mai 2006 (BGBl. I S. 1206, 1313), das zuletzt durch Artikel 280 der Verordnung vom 19. Juni 2020 (BGBl. I S. 1328) geändert worden ist.

[bib67] Touma C., Palme R. (2005). Measuring fecal glucocorticoid metabolites in mammals and birds: the importance of validation. Ann. N. Y. Acad. Sci..

[bib68] Tucker H.A., Schwalm J.W. (1977). Glucocorticoids in mammary Tissue and milk. J. Anim. Sci..

[bib69] von Eugen K., Nordquist R.E., Zeinstra E., van der Staay F.J. (2019). Stocking density affects stress and anxious behavior in the laying hen chick during rearing. Animals.

[bib70] Weimer S.L., Wideman R.F., Scanes C.G., Mauromoustakos A., Christensen K.D., Vizzier-Thaxton Y. (2018). An evaluation of methods for measuring stress in broiler chickens. Poult. Sci..

[bib71] Zeinstra E.C., Oeben L., van der Staay F.J., Nordquist R.E. (2015). Methoden voor de bepaling van corticosteron in veren en cortisol en haren als mogelijke lange-termijn indicator voor dierenwelzijn. Biotechniek.

